# The *Chlorella vulgaris S*-Nitrosoproteome under Nitrogen-Replete and -Deplete Conditions

**DOI:** 10.3389/fbioe.2016.00100

**Published:** 2017-01-17

**Authors:** Calvin A. Henard, Michael T. Guarnieri, Eric P. Knoshaug

**Affiliations:** ^1^National Bioenergy Center, National Renewable Energy Laboratory, Golden, CO, USA

**Keywords:** *Chlorella*, biofuels, microalgae, S-nitrosylation, nitric oxide

Oleaginous microalgae synthesize and accumulate large quantities of lipids that are promising feedstocks for the production of biofuels (Hu et al., 2008; Williams and Laurens, 2010; Day et al., 2012; Quinn and Davis, 2015). The algal species *Chlorella vulgaris* accumulates triacylglycerides that dominate its cellular composition (>60% lipid based on dry cell weight) when cultured in medium lacking a nitrogen source (Guarnieri et al., 2011; Ikaran et al., 2015), which is a “lipid trigger” in an array of microalgae. As such, *C. vulgaris* represents a model algal species for examination of lipid accumulation mechanisms and a potential deployment organism in industrial algal biofuels applications. *C. vulgaris* has been extensively characterized biochemically and physiologically (Converti et al., 2009; Liang et al., 2009), and *de novo*-generated transcriptomic and proteomic datasets have indicated that post-transcriptional and -translational mechanisms likely govern lipid accumulation in response to nitrogen starvation (Guarnieri et al., 2011, 2013). However, the specific mechanisms underlying lipid biosynthesis in response to nitrogen stress remain elusive.

Nitric oxide (NO) has received much attention as a signaling molecule due to its ability to react specifically with a limited number of biomolecules, primarily mediating physiological changes by modifying proteins in diverse domains of life, including plants, animals, and prokaryotes. This diatomic radical targets [Fe–S] clusters of dehydratases in several central metabolic pathways and can react with redox active sulfhydryls in cysteines to produce *S*-nitrosothiols. Protein *S*-nitrosylation by NO has been shown to play important roles in an array of cellular responses in organisms ranging from prokaryotes to metazoans. Previous studies have identified several proteins as targets of NO in higher plants, confirming S-nitrosylation as a key post-translational mechanism governing cell signaling in these organisms (Zaffagnini et al., 2016). Additional studies in the green microalga *Chlamydomonas reinhardtii* have identified hundreds of *S*-nitrosylated proteins after treatment with *S*-nitrosoglutathione (Samuel et al., 2014), supporting S-nitrosylation as a widespread post-translational mechanism employed by autotrophic organisms.

Based on the nitrate reductase-dependent NO production observed in higher plants and microalgae (Sakihama et al., 2002; Mur et al., 2013), we evaluated whether NO is produced by *C. vulgaris* when cultured in medium with nitrate as the sole nitrogen source. Using the NO-reactive fluorophore 4,5-diaminofluorescein, we detected NO produced by *C vulgaris* during logarithmic growth in modified Bold’s Basal Medium (BBM) supplemented with 3 mM sodium nitrate (Figure 1). The NO detected in algae exposed to nitrite in acidified BMM (pH 4.5), a condition that non-enzymatically generates high concentrations of NO congeners (Henard et al., 2014), was 10-fold higher than the concentration endogenously produced by this organism under nitrogen-replete growth (Figure 1A). NO was detected throughout the cell cytoplasm of cells grown in the presence of acidified nitrite, with more intense staining observed in the chloroplast (Figure 1B).

**Figure 1:**
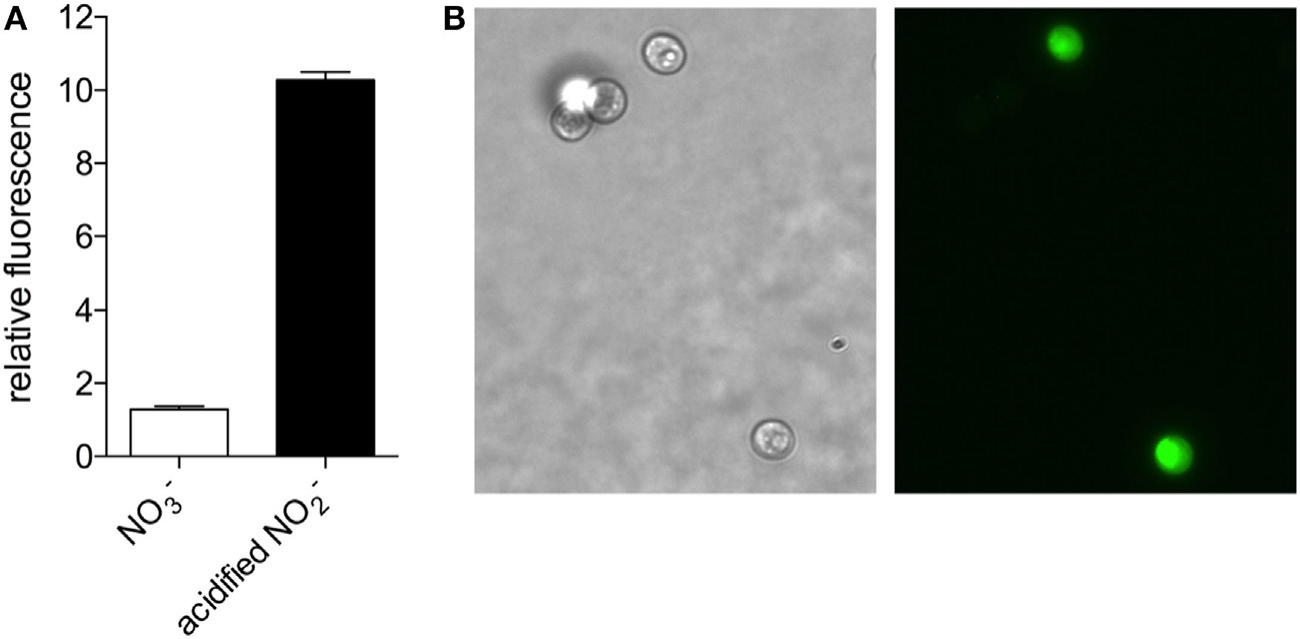
**Nitric oxide production in *Chlorella vulgaris* detected by 4,5-diaminofluorescein staining. (A)** *C. vulgaris* grown in Bold’s Basal Medium (BBM) with 3 mM NaNO_3_ (white bar) or treated with 1 mM NaNO_2_ in BBM pH 4.5 for 1 h (black bar) were stained with 4,5-diaminofluorescein to detect nitric oxide production fluorometrically. **(B)** Bright field (left panel) and fluorescence (right panel) microscopy of a representative NaNO_2_-treated sample.

To assess whether the nitrate-derived NO is biologically active, we determined the *S*-nitrosoproteome of *C. vulgaris* in the presence or absence of nitrate. The biotin switch method identified 40 proteins that are endogenously *S*-nitrosylated in *C. vulgaris* during active growth (Table 1, nitrogen replete). *S*-nitrosylated proteins were identified from a variety of metabolic pathways including photosynthesis and light capture, the Calvin–Benson cycle and carbon capture, protein synthesis, the TCA cycle, stress response, fatty acid biosynthesis, and cell structure. Several of the *S*-nitrosylated proteins identified here have confirmed orthologous targets of NO in diverse organisms, including plants and algae (Zaffagnini et al., 2016). Interestingly, 80% of the *S*-nitrosothiols detected during active growth were undetected under nitrogen starvation (Table 1, nitrogen deplete) with only 9 of the 40 proteins identified as being *S*-nitrosylated during nitrogen-replete conditions still identified during nitrogen starvation conditions. Collectively, the decrease in *S*-nitrosylated proteins identified under nitrogen starvation suggests S-nitrosylation occurs primarily during growth in a nitrogen-replete environment, presumably due to nitrate reductase-mediated NO production. Several proteins identified here have been previously identified as *S*-nitrosylated in plants, mammals, and prokaryotes in the presence of exogenous NO donors; however, our data represent targets *S*-nitrosylated by endogenously produced NO *via* nitrate metabolism.

**Table 1:** ***Chlorella vulgaris S*-nitrosylated proteins under nitrate-replete and -deplete conditions**.

				Replete		Deplete		
Scaffold.gene	BLASTP match	*e* Value	Molecular weight (kDa)	# unique peptides	% coverage	# unique peptides	% coverage	# cysteine residues
**Photosynthesis**								
291.25	Photosystem I subunit VII	3.80E−52	9	3	38			9
432.15	Chlorophyll a/b-binding protein	1.00E−152	27	4	42			2
772.94	Chloroplast light-harvesting complex II protein precursor	0.00E+00	52	7	28	4	8	2
1102.1	Photosystem I p700 chlorophyll a apoprotein a1	0.00E+00	83	3	3	2	2	4
1102.11	Photosystem I p700 apoprotein a2	0.00E+00	70	3	5			3
1102.7	Photosystem II 47 kDa protein	0.00E+00	56	5	8			3
1280.117	Photosystem I subunit chloroplast precursor	8.20E−90	20	2	11			3
1313.7	Photosystem I light-harvesting protein	5.20E−133	28	4	18			3
1313.76	Photosystem I light-harvesting protein	1.80E−130	23	2	11			1
1504.25	Chlorophyll a/b binding	2.30E−158	31	2	6			1
1611.13	Photosystem I subunit XI precursor	5.20E−98	46	2	7			11
1615.55	Porphobilinogen deaminase	0.00E+00	38					5
1759.19	Photosystem I light-harvesting protein	1.10E−126	79	2	2			5
2265.19	Photosystem II oxygen-evolving complex	2.10E−81	26	2	10			0
2779.8	Light-harvesting chlorophyll a/b-binding protein	1.20E−155	27	4	19	3	15	1
**Calvin–Benson cycle/carbon capture and concentration**								
101.72	Phosphoglycerate kinase	0.00E+00	49	3	8			3
669.32	Glyceraldehyde-3-phosphate dehydrogenase	0.00E+00	43	8	24			4
971.4	Low-CO_2_ inducible protein	0.00E+00	55	6	22	3	12	10
1137.114	Transketolase	0.00E+00	77	3	5			9
1250.71	Glyceraldehyde-3-phosphate dehydrogenase type I	0.00E+00	38	2	9	2	9	5
1605.9	Glyceraldehyde-3-phosphate dehydrogenase type I	8.60E−125	22	5	33	2	12	2
1655.2	Ribulose bisphosphate small subunit precursor	7.60E−84	15	5	44	2	20	4
1691.8	Fructose bisphosphate aldolase	0.00E+00	41	6	18			4
**Protein synthesis**								
352.92	Glutamine synthetase	0.00E+00	42	2	8			10
355.1	Elongation factor tu	0.00E+00	45	3	10			2
407.59	Choline dehydrogenase	0.00E+00	64	2	4			7
564.107	60S ribosomal protein	4.30E−107	17	2	17			3
759.8	Eukaryotic translation elongation factor 1 alpha 2	0.00E+00	49	16	39	7	17	10
835.5	Carbamoyl-phosphate synthase	0.00E+00	117	2	2			17
1136.46	Elongation factor 2	0.00E+00	68	2	4			16
1412.67	Homocysteine *S*-methyltransferase	0.00E+00	109	2	4			20
2123.11	Glutamate-1-semialdehyde-aminomutase	0.00E+00	47	3	10			7
**TCA cycle/energy generation**								
291.19	ATP synthase CF1-beta subunit	0.00E+00	49	5	18			3
291.6	ATP synthase CF1 alpha subunit	0.00E+00	55	4	11			1
1889.22	Dihydrolipoyl (dihydrolipamide) dehydrogenase	0.00E+00	53	2	5			6
**Stress response/signaling cascades**								
545.25	l-Ascorbate peroxidase	1.20E−153	43	2	8			9
1123.1	Calcium calmodulin-dependent protein kinase	3.50E−100	25	2	13			1
1196.84	Hop-interacting protein thi045	1.80E−110	25	8	45	3	18	2
**Fatty acid biosynthesis**								
2978.6	Biotin carboxylase	2.40E−164	31	2	10			2
**Cell maintenance and growth**								
439.32	Beta-tubulin	0.00E+00	47	3	8			13

S-nitrosylation of proteins identified here could have direct roles in lipid accumulation in *C. vulgaris*. The differential S-nitrosylation of biotin carboxylase observed during nitrogen-deplete and -replete conditions may play a direct role in *C. vulgaris* lipid accumulation. Biotin carboxylase is a subunit of the acetyl-CoA carboxylase (ACCase) complex and production of malonyl-CoA by this enzyme complex is considered the first committing step in lipid triacylglycerol (TAG) biosynthesis. We previously noted that this enzyme is upregulated during nitrogen starvation (Guarnieri et al., 2013), but, interestingly, we only detected *S*-nitrosylated biotin carboxylase during growth in the presence of nitrate. We should note that since biotin is a cofactor utilized by this enzyme, it is possible that even in the absence of being *S*-nitrosylated, it can potentially bind to the streptavidin matrix and be enriched with *S*-nitrosothiols during the biotin switch assay. However, although biotin carboxylase is upregulated during nitrogen starvation, we did not detect this enzyme under nitrogen-deplete conditions, indicating that its enrichment in nitrogen-replete samples was not simply biotinylated enzyme. Given the low level of neutral lipids produced during nitrogen-replete growth, biotin carboxylase represents a potential protein-engineering target to improve TAG accumulation during active growth.

Dihydrolipoyl dehydrogenase (DHLD) functions as a subunit in the α-ketoacid dehydrogenase complexes. As a subunit in the pyruvate dehydrogenase complex, DHLD is involved in converting pyruvate to acetyl-CoA and has been implemented in increasing lipid production by providing additional lipid precursors (acetyl-CoA) in *Chlorella protothecoides* grown heterotrophically on sugarcane bagasse hydrolyzate (Mu et al., 2015). DHLD has also been shown to be *S*-nitrosylated in plants responding to pathogens, cold, and abiotic stress (Maldonado-Alconada et al., 2011; Ortega-Galisteo et al., 2012; Puyaubert et al., 2014). As with biotin carboxylase, DHLD is only *S*-nitrosylated during nitrogen-replete growth and represents another potential target for protein engineering to eliminate the negative effects of S-nitrosylation on this enzyme.

Six enzymes involved in the Calvin cycle were identified as *S*-nitrosylated including ribulose bisphosphate carboxylase/oxygenase, phosphoglycerate kinase, three isozymes of glyceraldehyde-3-phosphate (GAPDH), and fructose bisphoshate aldolase (FBA). GAPDH has been previously reported to be *S*-nitrosylated in plants responding to cold, ozone, high light, salt, or pathogen-induced stress (Maldonado-Alconada et al., 2011; Astier and Lindermayr, 2012; Puyaubert et al., 2014; Vanzo et al., 2014). GAPDH regulation in response to nitrogen stress in *C. vulgaris* appears to be quite complex as its three isozymes are differentially regulated, one is downregulated, while the other two are upregulated during nitrogen stress (Guarnieri et al., 2013). Interestingly, S-nitrosylation of GAPDH has been shown to inhibit this enzyme and cause a shift in its activity where it localizes to the nucleus and acts as an *S*-nitrosylase and *S*-nitrosylates other target proteins (Astier and Lindermayr, 2012; Zaffagnini et al., 2013). Thus, *S*-nitrosylated GAPDH could mediate global transcriptional and metabolic regulatory alterations in *C. vulgaris*. We also identified FBA as *S*-nitrosylated under nitrogen-replete growth. In *Arabidopsis*, FBA is important for modulating stress responses and is inhibited by S-nitrosylation (van der Linde et al., 2011; Lu et al., 2012). Together with the inhibition of GAPDH, FBA inhibition could result in increased carbon flux through the oxidative branch of the pentose phosphate pathway under nitrogen-replete growth.

These data support endogenously produced NO during nitrate metabolism as a potential signaling molecule and indicate that protein S-nitrosylation could be a common post-translational modification utilized by *C. vulgaris* to regulate responses to nitrogen availability. Interestingly, a significant percentage (30%) of the *S*-nitrosylated proteins are differentially regulated under nitrogen-replete and nitrogen-deplete conditions (Guarnieri et al., 2013), suggesting that NO might be a key post-translational regulator of lipid biosynthesis in *C. vulgaris*. Collectively, the data presented here provide insight into the physiological role of S-nitrosylation as it relates to lipid accumulation, which can inform *C. vulgaris* engineering strategies to enable algae-based biofuels.

## Materials and Methods

### Culture Conditions and Biotin Switch

*Chlorella vulgaris* UTEX395 was grown in BBM supplemented with 3 mM NaNO_3_ to an OD_750nm_ of 2 as previously described (Guarnieri et al., 2011). Equal concentrations of logarithmically growing cells were harvested, washed in nitrate-free BBM, suspended in BBM with or without 3 mM NaNO_3_ to OD_750nm_ = 2.0, and grown in 500 mL shake flasks. After 24 h of incubation, whole cell lysates were prepared and enriched for *S*-nitrosylated proteins using the biotin switch pull-down assay as previously described (Jaffrey and Snyder, 2001; Forrester et al., 2009).

### Fluorometric Detection of NO

*Chlorella vulgaris* grown in BBM with 3 mM NaNO_3_ to an OD_750nm_ of 2 were pelleted, washed 2× in nitrate-free BMM, and resuspended at a concentration of 1 × 10^6^ cells/mL in BBM pH 4.5 supplemented with 1 mM NaNO_2_ or NaNO_3_ for 1 h. After treatment, equal concentrations of NaNO_2_-treated, NaNO_3_-treated, and untreated cells were stained with 4,5-diaminofluorescein (Cayman Chemical, Ann Arbor, MI, USA) to detect nitric oxide production fluorometrically.

### Mass Spectrometry Analysis

Peptides were purified and concentrated using an online enrichment column (Thermo Scientific 5 µm, 100 µm ID × 2 cm C18 column). Subsequent chromatographic separation was performed on a reverse phase nanospray column (Thermo Scientific EASYnano-LC, 3 µm, 75 µm ID × 100 mm C18 column) using a 30 min linear gradient from 10–30% buffer B (100% ACN, 0.1% formic acid) at a flow rate of 400 nL/min. Peptides were eluted directly into the mass spectrometer (Thermo Scientific Orbitrap Velos) and spectra were collected over a *m*/*z* range of 400–2000 Da using a dynamic exclusion limit of two MS/MS spectra of a given peptide mass for 30 s (exclusion duration of 90 s). The instrument was operated in Orbitrap-LTQ mode where precursor measurements were acquired in the orbitrap (60,000 resolution) and MS/MS spectra (top 20) were acquired in the LTQ ion trap with a normalized collision energy of 35 kV. Compound lists of the resulting spectra were generated using Xcalibur 2.2 software (Thermo Scientific) with an *S*/*N* threshold of 1.5 and 1 scan/group.

### Protein Identification

Tandem mass spectra were extracted, charge state deconvoluted and deisotoped by ProteoWizard (MS-Convert) version 3.0. All MS/MS samples were analyzed using Mascot (Matrix Science, London, UK; version 2.3.02). Mascot was set up to search the Cv395_Maker_7100_peps database (unknown version, 7,100 entries) assuming the digestion enzyme trypsin. Mascot was searched with a fragment ion mass tolerance of 0.80 Da and a parent ion tolerance of 20 ppm. Oxidation of methionine and carbamidomethyl of cysteine were specified in Mascot as variable modifications. Scaffold (version Scaffold_4.3.2, Proteome Software Inc., Portland, OR, USA) was used to validate MS/MS-based peptide and protein identifications. Peptide identifications were accepted if they could be established at greater than 85.0% probability by the Peptide Prophet algorithm (Keller et al., 2002) with Scaffold delta-mass correction. Protein identifications were accepted if they could be established at greater than 80.0% probability and contained at least one identified peptide. Protein probabilities were assigned by the Protein Prophet algorithm (Nesvizhskii et al., 2003). Proteins that contained similar peptides and could not be differentiated based on MS/MS analysis alone were grouped to satisfy the principles of parsimony. All identified peptides had a >95% probability of correct identification based on a BLASTP search of the predicted peptides against the predicted proteins present in the draft *C. vulgaris* genome (Accession#: LDKB01000000). *S*-nitrosylated targets identified here under nitrogen-deplete and -replete conditions are presented in Table 1 and deposited as an excel file at Figshare at the following link: https://figshare.com/s/39ea5540257b0efa9d5d.

### Quality Control

Instrument functionality and stability was monitored using the MassQC software (Proteome Software). This software uses a set of quantitative metrics developed by the National Institute of Science and Technology in collaboration with the National Cancer Institute’s Clinical Proteomic Technologies for Cancer (CPTC) that monitor technical variability in mass spectrometry-based proteomics instrumentation. Quality control samples containing a mixture of six trypsin digested bovine proteins were injected at least once every 24 h throughout the analysis, and the data from this run were analyzed using the MassQC software. Values for all metrics were within normal limits throughout the duration of the experiment indicating instrument stability and data robustness.

## Author Contributions

CH and EK designed experimental strategies. CH performed experiments and CH, MG, and EK analyzed data. CH, MG, and EK wrote the manuscript.

## Conflict of Interest Statement

The authors declare that the research was conducted in the absence of any commercial or financial relationships that could be construed as a potential conflict of interest.
